# 3,5-Dimethyl-1-{2-[(5-methyl-1,3,4-thia­diazol-2-yl)sulfan­yl]acet­yl}-2,6-diphenylpiperidin-4-one

**DOI:** 10.1107/S1600536813012014

**Published:** 2013-05-11

**Authors:** S. Ganesan, P. Sugumar, S. Ananthan, M. N. Ponnuswamy

**Affiliations:** a Research Development Centre, Orchid Chemicals and Pharmaceuticals Ltd, Sozhinganallur, Chennai 600 119, India; b Department of Chemistry, Presidency College (Autonomous), Chennai 600 005, India; c Centre of Advanced Study in Crystallography and Biophysics, University of Madras, Guindy Campus, Chennai 600 025, India

## Abstract

In the title compound, C_24_H_25_N_3_O_2_S_2_, the piperidine ring adopts a distorted boat conformation. The phenyl rings subtend angles of 75.6 (1)° and 86.3 (1)° with the mean plane of the piperidine ring. In the crystal, mol­ecules are linked through a network C—H⋯N hydrogen bonds, forming zigzag chains along [100]. The thia­diazol ring methyl group is disordered over two positions with an occupancy ratio of 0.69 (4):0.31 (4).

## Related literature

For the biological activity of piperidine derivatives, see: Aridoss *et al.* (2009 ▶). For puckering parameters, see: Cremer & Pople (1975 ▶) and for asymmetry parameters, see: Nardelli (1983 ▶). For hydrogen-bond motifs, see: Bernstein *et al.*(1995 ▶).
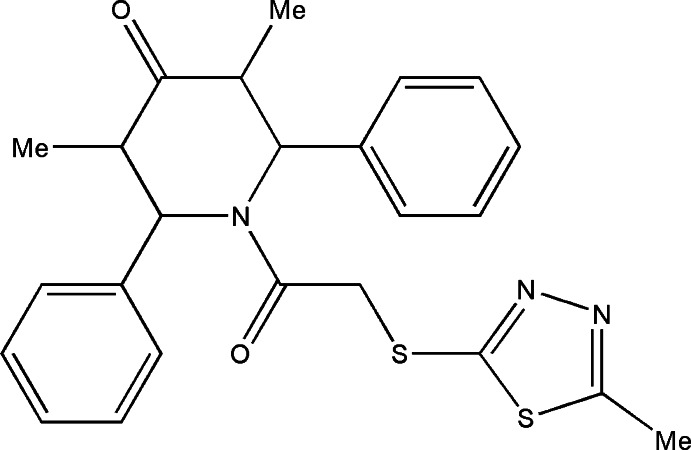


## Experimental

### 

#### Crystal data

C_24_H_25_N_3_O_2_S_2_
*M* *_r_* = 451.59
Orthorhombic, 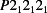

*a* = 9.1342 (6) Å
*b* = 9.2874 (6) Å
*c* = 27.0454 (14) Å
*V* = 2294.3 (2) Å^3^
*Z* = 4
Mo *K*α radiation
μ = 0.26 mm^−1^
*T* = 293 K
0.30 × 0.30 × 0.25 mm

#### Data collection

Bruker SMART APEXII CCD diffractometer
Absorption correction: multi-scan (*SADABS*; Bruker, 2008 ▶) *T*_min_ = 0.927, *T*_max_ = 0.938
10655 measured reflections
4215 independent reflections
3623 reflections with *I* > 2σ(*I*)
*R*_int_ = 0.033

#### Refinement

*R*[*F* ^2^ > 2σ(*F* ^2^)] = 0.040
*wR*(*F* ^2^) = 0.098
*S* = 1.06
4215 reflections
293 parameters
2 restraints
H-atom parameters constrained
Δρ_max_ = 0.18 e Å^−3^
Δρ_min_ = −0.24 e Å^−3^
Absolute structure: Flack (1983 ▶), 1461 Friedel pairs
Flack parameter: 0.26 (8)

### 

Data collection: *APEX2* (Bruker, 2008 ▶); cell refinement: *SAINT* (Bruker, 2008 ▶); data reduction: *SAINT*; program(s) used to solve structure: *SHELXS97* (Sheldrick, 2008 ▶); program(s) used to refine structure: *SHELXL97* (Sheldrick, 2008 ▶); molecular graphics: *ORTEP-3 for Windows* (Farrugia, 2012 ▶); software used to prepare material for publication: *SHELXL97* and *PLATON* (Spek, 2009 ▶).

## Supplementary Material

Crystal structure: contains datablock(s) global, I. DOI: 10.1107/S1600536813012014/ng5326sup1.cif

Structure factors: contains datablock(s) I. DOI: 10.1107/S1600536813012014/ng5326Isup2.hkl

Supplementary material file. DOI: 10.1107/S1600536813012014/ng5326Isup3.cml

Additional supplementary materials:  crystallographic information; 3D view; checkCIF report

## Figures and Tables

**Table 1:** Hydrogen-bond geometry (Å, °)

*D*—H⋯*A*	*D*—H	H⋯*A*	*D*⋯*A*	*D*—H⋯*A*
C6—H6⋯N3^i^	0.98	2.48	3.460 (4)	175
C22—H22*B*⋯N2^i^	0.97	2.47	3.403 (4)	161
C22—H22*B*⋯N3^i^	0.97	2.57	3.505 (4)	161

Symmetry code: (i) 
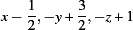
.

**Fig. 1.**
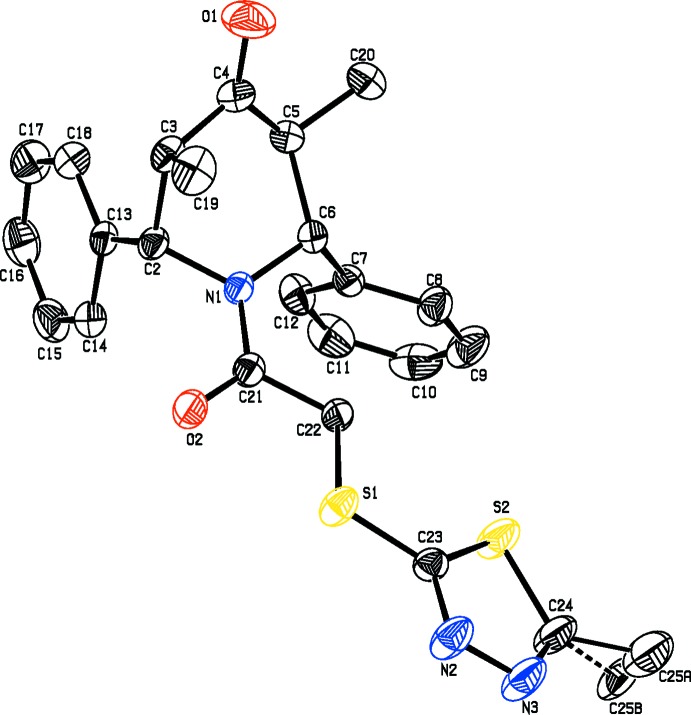
The molecular structure of the title compound, showing the atomic numbering and displacement ellipsoids drawn at 30% probability level.

**Fig. 2.**
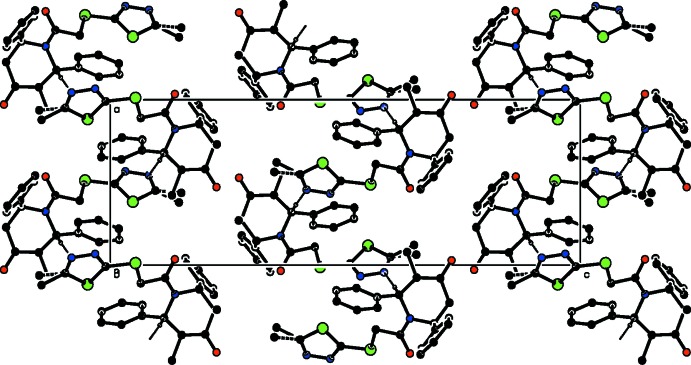
The crystal packing of the molecules viewed down b axis.

**Fig. 3.**
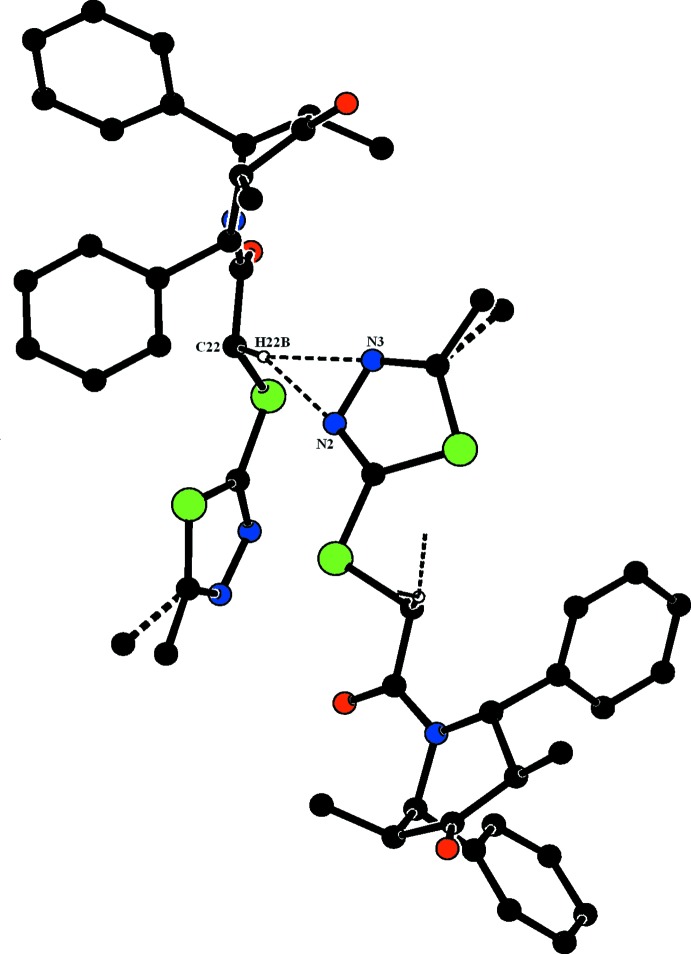
The C–H···N interactions constitute a pair of bifurcated donor bonds.

## supplementary crystallographic information

### Comment

In a way to find piperidin-4-one based lead drug molecules for the antimicrobial therapy, a new series of piperidin-4-one derivatives were prepared by condensing *N*-Chloroacetyl-2,6-diphenylpiperidin-4-one with 5-Methyl-1,3,4-thiadiazole-2-thiol. Report suggests that the substitution at chloro position of *N*-chloroacetyl-2,6-diphenylpiperidin-4-one alters the activity of parent compounds (Aridoss *et al.*, 2009). 5-Methyl-1,3,4-thiadiazole-2-thiol is part of a number of cephalosporanic drugs *viz* Cefazolin and responsible for its activity. Keeping these two facts in mind, we have prepared a series of piperidin-4-ones. The present investigation was undertaken to establish the molecular structure and conformation of the title compound by X-ray diffraction method.

The *ORTEP* plot of the molecule is shown in Fig. 1. The piperidine ring adopts distorted boat conformation with the puckering parameters (Cremer & Pople, 1975) and the asymmetry parameters (Nardelli,1983) are: q_2_=0.663 (3) Å, q_3_ = -0.045 (3) Å, φ_2_ = 245.0 (2)° and Δ_s_(N1 & C4)= 68.3 (2)°.

The heterocyclic 1,3,4-thiadiazole ring is planar with the maximum deviation of atom C24 is -0.007 (4)°. The bond lengths of the endocyclic bonds [C23–N2 = 1.280 (4) Å & C24–N3 = 1.270 (5) Å], clearly indicate that they are double bond in character. The methyl group substituted at C25 atom in thiadiazol ring is disordered over two positions with the site occupancy of 0.69 (4) and 0.31 (4).

The carbonyl group is oriented *anti-periplanar* to C2 [C2—C3—C4—O1=] -171.9 (3)° and *anti-clinal* to C6 [C6—C5—C4—O1=] -139.1 (3)°. The best plane of the piperidine ring and the attached phenyl rings [C7—C12 & C13—C18] are twisted away by 75.6 (1)° and 86.3 (1)°. The two phenyl rings are oriented to each other with a dihedral angle of 59.7 (1)°.

The crystal packing reveals that the symmetry related molecules are linked through a network of C—H···O, C—H···N & C—H···S types of intra and intermolecular interactions. The hydrogen bonded network play a role in stabilizing the molecules in the unit cell (Fig. 2). Interesting to note that the C22—H22B···N3 and C22—H22B···N2 interactions together constitute a pair of bifurcated donor bonds as shown in Fig. 3 (Bernstein *et al.*, 1995).

### Experimental

To anhydrous DMF (10 ml), *N*-chloroacetyl-3,5-dimethyl-2,6-diphenylpiperidin-4-one (1 mole), 5-Methyl-1,3,4-thiadiazole-2-thiol(1 mole) followed by potassium carbonate (1.5 mole) was added and stirred for 1 hr at room temperature. The reaction mass was heated to 60° C and stirred. The reaction was monitored using TLC. After completion of reaction, the reaction mass was quenched into water and the product was extracted with dichloromethane. The dichloromethane layer distilled completely and to the residue methanol was added and kept overnight. The solid obtained was filtered and dried at 60° C under vacuum. Single crystal was obtained by re-crystallization using ethanol.

### Refinement

N and C-bound H atoms were positioned geometrically (C–H = 0.93–0.98 Å) and allowed to ride on their parent atoms, with *U*_iso_(H) = 1.5*U*_eq_(C) for methyl H atoms and 1.2*U*_eq_(C) for all other H atoms. The methyl atom (C25) is disordered over two set of positions with refined site-occupancies ratio of 0.69 (4)/0.31 (4).

### Crystal data

**Table:**

C_24_H_25_N_3_O_2_S_2_	*F*(000) = 952
*M**_r_* = 451.59	*D*_x_ = 1.307 Mg m^−^^3^
Orthorhombic, *P*2_1_2_1_2_1_	Mo *K*α radiation, λ = 0.71073 Å
Hall symbol: P 2ac 2ab	Cell parameters from 4138 reflections
*a* = 9.1342 (6) Å	θ = 1.5–28.4°
*b* = 9.2874 (6) Å	µ = 0.26 mm^−^^1^
*c* = 27.0454 (14) Å	*T* = 293 K
*V* = 2294.3 (2) Å^3^	Block, white crystalline
*Z* = 4	0.30 × 0.30 × 0.25 mm

### Data collection

**Table:**

Bruker SMART APEXII CCD diffractometer	4215 independent reflections
Radiation source: fine-focus sealed tube	3623 reflections with *I* > 2σ(*I*)
Graphite monochromator	*R*_int_ = 0.033
ω and φ scans	θ_max_ = 25.5°, θ_min_ = 1.5°
Absorption correction: multi-scan (*SADABS*; Bruker, 2008)	*h* = −11→10
*T*_min_ = 0.927, *T*_max_ = 0.938	*k* = −10→11
10655 measured reflections	*l* = −32→32

### Refinement

**Table:**

Refinement on *F*^2^	Secondary atom site location: difference Fourier map
Least-squares matrix: full	Hydrogen site location: inferred from neighbouring sites
*R*[*F*^2^ > 2σ(*F*^2^)] = 0.040	H-atom parameters constrained
*wR*(*F*^2^) = 0.098	*w* = 1/[σ^2^(*F*_o_^2^) + (0.0407*P*)^2^ + 0.4037*P*] where *P* = (*F*_o_^2^ + 2*F*_c_^2^)/3
*S* = 1.06	(Δ/σ)_max_ = 0.003
4215 reflections	Δρ_max_ = 0.18 e Å^−^^3^
293 parameters	Δρ_min_ = −0.24 e Å^−^^3^
2 restraints	Absolute structure: Flack (1983), 1461 Friedel pairs
Primary atom site location: structure-invariant direct methods	Flack parameter: 0.26 (8)

### Special details

**Table:**

Geometry. All e.s.d.'s (except the e.s.d. in the dihedral angle between two l.s. planes) are estimated using the full covariance matrix. The cell e.s.d.'s are taken into account individually in the estimation of e.s.d.'s in distances, angles and torsion angles; correlations between e.s.d.'s in cell parameters are only used when they are defined by crystal symmetry. An approximate (isotropic) treatment of cell e.s.d.'s is used for estimating e.s.d.'s involving l.s. planes.
Refinement. Refinement of *F*^2^ against ALL reflections. The weighted *R*-factor *wR* and goodness of fit *S* are based on *F*^2^, conventional *R*-factors *R* are based on *F*, with *F* set to zero for negative *F*^2^. The threshold expression of *F*^2^ > σ(*F*^2^) is used only for calculating *R*-factors(gt) *etc*. and is not relevant to the choice of reflections for refinement. *R*-factors based on *F*^2^ are statistically about twice as large as those based on *F*, and *R*- factors based on ALL data will be even larger.

### Fractional atomic coordinates and isotropic or equivalent isotropic displacement parameters (Å^2^)

**Table:**

	*x*	*y*	*z*	*U*_iso_*/*U*_eq_	Occ. (<1)
C2	0.8537 (3)	0.3491 (3)	0.31092 (8)	0.0434 (6)	
H2	0.9412	0.4016	0.3003	0.052*	
C3	0.7285 (3)	0.4013 (3)	0.27855 (9)	0.0498 (6)	
H3	0.7412	0.3594	0.2456	0.060*	
C4	0.5804 (3)	0.3549 (3)	0.29802 (10)	0.0557 (7)	
C5	0.5761 (3)	0.2856 (3)	0.34821 (9)	0.0468 (6)	
H5	0.6113	0.1866	0.3442	0.056*	
C6	0.6804 (2)	0.3605 (3)	0.38547 (8)	0.0409 (5)	
H6	0.6351	0.4523	0.3948	0.049*	
C7	0.6940 (2)	0.2704 (3)	0.43237 (9)	0.0420 (6)	
C8	0.6392 (3)	0.3216 (3)	0.47632 (9)	0.0565 (7)	
H8	0.5926	0.4105	0.4773	0.068*	
C9	0.6530 (4)	0.2415 (4)	0.51908 (11)	0.0761 (10)	
H9	0.6165	0.2776	0.5487	0.091*	
C10	0.7197 (4)	0.1100 (4)	0.51842 (13)	0.0766 (10)	
H10	0.7295	0.0570	0.5474	0.092*	
C11	0.7717 (4)	0.0571 (4)	0.47490 (13)	0.0716 (9)	
H11	0.8162	−0.0329	0.4741	0.086*	
C12	0.7588 (3)	0.1361 (3)	0.43181 (10)	0.0559 (7)	
H12	0.7941	0.0986	0.4022	0.067*	
C13	0.8916 (3)	0.1899 (3)	0.30846 (8)	0.0431 (6)	
C14	1.0060 (3)	0.1415 (3)	0.33779 (9)	0.0569 (7)	
H14	1.0598	0.2074	0.3562	0.068*	
C15	1.0411 (3)	−0.0013 (4)	0.34010 (12)	0.0689 (9)	
H15	1.1161	−0.0316	0.3609	0.083*	
C16	0.9671 (4)	−0.1006 (3)	0.31216 (12)	0.0705 (9)	
H16	0.9906	−0.1978	0.3143	0.085*	
C17	0.8584 (4)	−0.0551 (3)	0.28114 (12)	0.0667 (8)	
H17	0.8097	−0.1209	0.2611	0.080*	
C18	0.8208 (3)	0.0898 (3)	0.27956 (10)	0.0535 (7)	
H18	0.7463	0.1197	0.2585	0.064*	
C19	0.7301 (4)	0.5638 (3)	0.27324 (12)	0.0756 (9)	
H19A	0.8236	0.5941	0.2608	0.113*	
H19B	0.7130	0.6073	0.3049	0.113*	
H19C	0.6546	0.5929	0.2506	0.113*	
C20	0.4220 (3)	0.2764 (4)	0.36868 (12)	0.0714 (9)	
H20A	0.4243	0.2312	0.4006	0.107*	
H20B	0.3621	0.2205	0.3467	0.107*	
H20C	0.3820	0.3715	0.3717	0.107*	
C21	0.9216 (3)	0.4863 (3)	0.38323 (9)	0.0447 (6)	
C22	0.8900 (3)	0.5401 (3)	0.43507 (9)	0.0444 (6)	
H22A	0.9070	0.4641	0.4590	0.053*	
H22B	0.7889	0.5711	0.4377	0.053*	
C23	0.9909 (3)	0.7090 (3)	0.50966 (9)	0.0471 (6)	
C24	0.9507 (4)	0.7112 (5)	0.59575 (11)	0.0869 (12)	
N1	0.8240 (2)	0.3941 (2)	0.36250 (7)	0.0391 (4)	
N2	1.0669 (3)	0.8047 (3)	0.53206 (9)	0.0749 (8)	
N3	1.0440 (3)	0.8048 (3)	0.58237 (9)	0.0874 (10)	
O1	0.4720 (3)	0.3740 (3)	0.27404 (9)	0.1004 (9)	
O2	1.0350 (2)	0.5239 (2)	0.36260 (7)	0.0672 (6)	
S1	1.01181 (9)	0.68920 (7)	0.44633 (2)	0.0565 (2)	
S2	0.88298 (10)	0.60962 (11)	0.54817 (3)	0.0817 (3)	
C25A	0.884 (3)	0.715 (3)	0.6474 (4)	0.123 (7)	0.69 (4)
H25A	0.8855	0.6198	0.6613	0.185*	0.69 (4)
H25B	0.7853	0.7490	0.6455	0.185*	0.69 (4)
H25C	0.9407	0.7784	0.6680	0.185*	0.69 (4)
C25B	0.951 (3)	0.649 (3)	0.6513 (6)	0.076 (6)	0.31 (4)
H25D	1.0483	0.6177	0.6598	0.113*	0.31 (4)
H25E	0.8853	0.5682	0.6534	0.113*	0.31 (4)
H25F	0.9202	0.7224	0.6738	0.113*	0.31 (4)

### Atomic displacement parameters (Å^2^)

**Table:**

	*U*^11^	*U*^22^	*U*^33^	*U*^12^	*U*^13^	*U*^23^
C2	0.0469 (13)	0.0527 (15)	0.0307 (11)	−0.0076 (12)	0.0032 (10)	−0.0014 (10)
C3	0.0618 (16)	0.0515 (15)	0.0360 (13)	0.0021 (13)	−0.0031 (11)	−0.0025 (12)
C4	0.0517 (15)	0.0668 (18)	0.0485 (15)	0.0017 (13)	−0.0114 (12)	−0.0039 (13)
C5	0.0402 (13)	0.0516 (15)	0.0485 (14)	−0.0038 (11)	−0.0022 (11)	−0.0039 (12)
C6	0.0387 (12)	0.0446 (13)	0.0393 (12)	0.0024 (10)	0.0010 (10)	−0.0030 (11)
C7	0.0361 (12)	0.0488 (15)	0.0411 (13)	−0.0055 (11)	0.0021 (10)	−0.0002 (11)
C8	0.0613 (17)	0.0627 (17)	0.0456 (15)	−0.0139 (14)	0.0082 (13)	−0.0046 (14)
C9	0.088 (2)	0.100 (3)	0.0400 (15)	−0.036 (2)	0.0062 (15)	−0.0004 (18)
C10	0.074 (2)	0.093 (3)	0.063 (2)	−0.032 (2)	−0.0199 (17)	0.030 (2)
C11	0.0616 (19)	0.071 (2)	0.082 (2)	0.0007 (16)	−0.0033 (17)	0.0289 (19)
C12	0.0499 (14)	0.0580 (16)	0.0597 (17)	0.0043 (13)	0.0076 (13)	0.0088 (14)
C13	0.0428 (13)	0.0522 (14)	0.0344 (11)	−0.0030 (12)	0.0071 (10)	−0.0059 (11)
C14	0.0457 (14)	0.0730 (18)	0.0520 (15)	0.0061 (15)	0.0007 (13)	−0.0141 (13)
C15	0.0548 (18)	0.083 (2)	0.0684 (19)	0.0264 (17)	0.0023 (15)	−0.0010 (17)
C16	0.069 (2)	0.0571 (17)	0.085 (2)	0.0112 (17)	0.0170 (17)	−0.0013 (17)
C17	0.072 (2)	0.0482 (16)	0.080 (2)	−0.0047 (15)	0.0036 (17)	−0.0152 (15)
C18	0.0551 (16)	0.0556 (16)	0.0499 (15)	−0.0007 (13)	−0.0023 (12)	−0.0042 (13)
C19	0.096 (3)	0.0586 (18)	0.072 (2)	0.0069 (18)	0.0009 (19)	0.0069 (17)
C20	0.0438 (15)	0.100 (3)	0.0701 (19)	−0.0074 (16)	0.0006 (14)	−0.0088 (19)
C21	0.0460 (14)	0.0477 (14)	0.0404 (13)	−0.0020 (12)	0.0018 (11)	−0.0019 (11)
C22	0.0424 (12)	0.0435 (13)	0.0474 (14)	−0.0041 (11)	0.0012 (11)	−0.0111 (11)
C23	0.0478 (13)	0.0401 (13)	0.0533 (14)	−0.0038 (12)	−0.0056 (12)	−0.0073 (11)
C24	0.085 (2)	0.128 (3)	0.0478 (16)	−0.058 (2)	0.0071 (16)	−0.0245 (19)
N1	0.0402 (10)	0.0430 (11)	0.0342 (9)	−0.0039 (9)	0.0017 (8)	−0.0036 (9)
N2	0.097 (2)	0.0737 (17)	0.0540 (14)	−0.0434 (16)	0.0062 (13)	−0.0155 (13)
N3	0.106 (2)	0.105 (2)	0.0513 (14)	−0.057 (2)	0.0087 (14)	−0.0253 (15)
O1	0.0660 (14)	0.159 (2)	0.0759 (14)	−0.0036 (16)	−0.0281 (12)	0.0246 (16)
O2	0.0582 (12)	0.0878 (14)	0.0554 (11)	−0.0297 (11)	0.0149 (10)	−0.0213 (10)
S1	0.0768 (5)	0.0470 (3)	0.0457 (3)	−0.0182 (3)	0.0007 (3)	−0.0046 (3)
S2	0.0872 (6)	0.1083 (7)	0.0495 (4)	−0.0573 (5)	0.0084 (4)	−0.0189 (4)
C25A	0.122 (10)	0.190 (16)	0.058 (4)	−0.076 (11)	0.007 (5)	−0.031 (6)
C25B	0.077 (11)	0.117 (14)	0.033 (5)	−0.028 (9)	0.015 (6)	−0.015 (6)

### Geometric parameters (Å, º)

**Table:**

C2—N1	1.481 (3)	C16—C17	1.367 (4)
C2—C3	1.519 (3)	C16—H16	0.9300
C2—C13	1.520 (3)	C17—C18	1.389 (4)
C2—H2	0.9800	C17—H17	0.9300
C3—C4	1.515 (4)	C18—H18	0.9300
C3—C19	1.516 (4)	C19—H19A	0.9600
C3—H3	0.9800	C19—H19B	0.9600
C4—O1	1.197 (3)	C19—H19C	0.9600
C4—C5	1.503 (4)	C20—H20A	0.9600
C5—C20	1.515 (4)	C20—H20B	0.9600
C5—C6	1.552 (3)	C20—H20C	0.9600
C5—H5	0.9800	C21—O2	1.227 (3)
C6—N1	1.484 (3)	C21—N1	1.357 (3)
C6—C7	1.525 (3)	C21—C22	1.516 (3)
C6—H6	0.9800	C22—S1	1.802 (2)
C7—C8	1.375 (4)	C22—H22A	0.9700
C7—C12	1.381 (4)	C22—H22B	0.9700
C8—C9	1.380 (4)	C23—N2	1.280 (3)
C8—H8	0.9300	C23—S2	1.705 (3)
C9—C10	1.365 (5)	C23—S1	1.733 (3)
C9—H9	0.9300	C24—N3	1.270 (4)
C10—C11	1.361 (5)	C24—C25A	1.523 (10)
C10—H10	0.9300	C24—C25B	1.61 (2)
C11—C12	1.382 (4)	C24—S2	1.711 (3)
C11—H11	0.9300	N2—N3	1.377 (3)
C12—H12	0.9300	C25A—H25A	0.9600
C13—C18	1.376 (4)	C25A—H25B	0.9600
C13—C14	1.387 (4)	C25A—H25C	0.9600
C14—C15	1.366 (4)	C25B—H25D	0.9600
C14—H14	0.9300	C25B—H25E	0.9600
C15—C16	1.370 (5)	C25B—H25F	0.9600
C15—H15	0.9300		
			
N1—C2—C3	108.3 (2)	C17—C16—C15	119.3 (3)
N1—C2—C13	110.96 (19)	C17—C16—H16	120.4
C3—C2—C13	117.2 (2)	C15—C16—H16	120.4
N1—C2—H2	106.6	C16—C17—C18	119.9 (3)
C3—C2—H2	106.6	C16—C17—H17	120.1
C13—C2—H2	106.6	C18—C17—H17	120.1
C4—C3—C19	108.9 (2)	C13—C18—C17	121.4 (3)
C4—C3—C2	112.4 (2)	C13—C18—H18	119.3
C19—C3—C2	111.4 (2)	C17—C18—H18	119.3
C4—C3—H3	108.0	C3—C19—H19A	109.5
C19—C3—H3	108.0	C3—C19—H19B	109.5
C2—C3—H3	108.0	H19A—C19—H19B	109.5
O1—C4—C5	122.1 (3)	C3—C19—H19C	109.5
O1—C4—C3	120.6 (3)	H19A—C19—H19C	109.5
C5—C4—C3	117.3 (2)	H19B—C19—H19C	109.5
C4—C5—C20	112.2 (2)	C5—C20—H20A	109.5
C4—C5—C6	112.2 (2)	C5—C20—H20B	109.5
C20—C5—C6	111.0 (2)	H20A—C20—H20B	109.5
C4—C5—H5	107.0	C5—C20—H20C	109.5
C20—C5—H5	107.0	H20A—C20—H20C	109.5
C6—C5—H5	107.0	H20B—C20—H20C	109.5
N1—C6—C7	113.06 (18)	O2—C21—N1	123.1 (2)
N1—C6—C5	111.39 (18)	O2—C21—C22	119.2 (2)
C7—C6—C5	110.1 (2)	N1—C21—C22	117.7 (2)
N1—C6—H6	107.3	C21—C22—S1	106.97 (17)
C7—C6—H6	107.3	C21—C22—H22A	110.3
C5—C6—H6	107.3	S1—C22—H22A	110.3
C8—C7—C12	118.5 (2)	C21—C22—H22B	110.3
C8—C7—C6	120.0 (2)	S1—C22—H22B	110.3
C12—C7—C6	121.4 (2)	H22A—C22—H22B	108.6
C7—C8—C9	120.3 (3)	N2—C23—S2	113.6 (2)
C7—C8—H8	119.8	N2—C23—S1	118.8 (2)
C9—C8—H8	119.8	S2—C23—S1	127.58 (15)
C10—C9—C8	120.8 (3)	N3—C24—C25A	120.8 (5)
C10—C9—H9	119.6	N3—C24—C25B	120.7 (8)
C8—C9—H9	119.6	N3—C24—S2	113.9 (2)
C11—C10—C9	119.3 (3)	C25A—C24—S2	124.0 (4)
C11—C10—H10	120.3	C25B—C24—S2	120.2 (8)
C9—C10—H10	120.3	C21—N1—C2	116.55 (19)
C10—C11—C12	120.6 (3)	C21—N1—C6	122.68 (19)
C10—C11—H11	119.7	C2—N1—C6	119.78 (18)
C12—C11—H11	119.7	C23—N2—N3	112.7 (2)
C7—C12—C11	120.4 (3)	C24—N3—N2	112.5 (2)
C7—C12—H12	119.8	C23—S1—C22	100.39 (12)
C11—C12—H12	119.8	C23—S2—C24	87.25 (14)
C18—C13—C14	117.4 (2)	C24—C25A—H25A	109.5
C18—C13—C2	125.1 (2)	C24—C25A—H25B	109.5
C14—C13—C2	117.5 (2)	C24—C25A—H25C	109.5
C15—C14—C13	121.2 (3)	C24—C25B—H25D	109.5
C15—C14—H14	119.4	C24—C25B—H25E	109.5
C13—C14—H14	119.4	H25D—C25B—H25E	109.5
C14—C15—C16	120.8 (3)	C24—C25B—H25F	109.5
C14—C15—H15	119.6	H25D—C25B—H25F	109.5
C16—C15—H15	119.6	H25E—C25B—H25F	109.5
			
N1—C2—C3—C4	−52.8 (3)	C13—C14—C15—C16	2.2 (5)
C13—C2—C3—C4	73.6 (3)	C14—C15—C16—C17	1.0 (5)
N1—C2—C3—C19	69.8 (3)	C15—C16—C17—C18	−2.2 (5)
C13—C2—C3—C19	−163.8 (2)	C14—C13—C18—C17	2.5 (4)
C19—C3—C4—O1	64.1 (4)	C2—C13—C18—C17	−177.1 (3)
C2—C3—C4—O1	−171.9 (3)	C16—C17—C18—C13	0.5 (5)
C19—C3—C4—C5	−115.6 (3)	O2—C21—C22—S1	15.4 (3)
C2—C3—C4—C5	8.4 (3)	N1—C21—C22—S1	−166.73 (18)
O1—C4—C5—C20	−13.2 (4)	O2—C21—N1—C2	−5.2 (4)
C3—C4—C5—C20	166.4 (3)	C22—C21—N1—C2	177.0 (2)
O1—C4—C5—C6	−139.0 (3)	O2—C21—N1—C6	−173.8 (2)
C3—C4—C5—C6	40.6 (3)	C22—C21—N1—C6	8.5 (3)
C4—C5—C6—N1	−43.1 (3)	C3—C2—N1—C21	−117.4 (2)
C20—C5—C6—N1	−169.6 (2)	C13—C2—N1—C21	112.6 (2)
C4—C5—C6—C7	−169.4 (2)	C3—C2—N1—C6	51.5 (3)
C20—C5—C6—C7	64.1 (3)	C13—C2—N1—C6	−78.4 (3)
N1—C6—C7—C8	121.4 (2)	C7—C6—N1—C21	−70.2 (3)
C5—C6—C7—C8	−113.2 (3)	C5—C6—N1—C21	165.2 (2)
N1—C6—C7—C12	−59.6 (3)	C7—C6—N1—C2	121.6 (2)
C5—C6—C7—C12	65.8 (3)	C5—C6—N1—C2	−3.0 (3)
C12—C7—C8—C9	1.9 (4)	S2—C23—N2—N3	0.2 (4)
C6—C7—C8—C9	−179.0 (2)	S1—C23—N2—N3	−177.5 (2)
C7—C8—C9—C10	−0.7 (5)	C25A—C24—N3—N2	−166.3 (15)
C8—C9—C10—C11	−0.6 (5)	C25B—C24—N3—N2	156.0 (14)
C9—C10—C11—C12	0.7 (5)	S2—C24—N3—N2	1.3 (5)
C8—C7—C12—C11	−1.8 (4)	C23—N2—N3—C24	−1.0 (5)
C6—C7—C12—C11	179.2 (3)	N2—C23—S1—C22	177.6 (2)
C10—C11—C12—C7	0.5 (5)	S2—C23—S1—C22	0.2 (2)
N1—C2—C13—C18	124.6 (2)	C21—C22—S1—C23	−167.14 (17)
C3—C2—C13—C18	−0.5 (3)	N2—C23—S2—C24	0.4 (3)
N1—C2—C13—C14	−55.0 (3)	S1—C23—S2—C24	177.8 (2)
C3—C2—C13—C14	179.8 (2)	N3—C24—S2—C23	−1.0 (3)
C18—C13—C14—C15	−3.8 (4)	C25A—C24—S2—C23	166.2 (16)
C2—C13—C14—C15	175.8 (3)	C25B—C24—S2—C23	−155.8 (14)

### Hydrogen-bond geometry (Å, º)

**Table:**

*D*—H···*A*	*D*—H	H···*A*	*D*···*A*	*D*—H···*A*
C6—H6···N3^i^	0.98	2.48	3.460 (4)	175
C22—H22*B*···N2^i^	0.97	2.47	3.403 (4)	161
C22—H22*B*···N3^i^	0.97	2.57	3.505 (4)	161

Symmetry code: (i) *x*−1/2, −*y*+3/2, −*z*+1.
